# Oxidative stress, innate immunity, and age-related macular degeneration

**DOI:** 10.3934/molsci.2016.2.196

**Published:** 2016-05-11

**Authors:** Peter X. Shaw, Travis Stiles, Christopher Douglas, Daisy Ho, Wei Fan, Hongjun Du, Xu Xiao

**Affiliations:** 1 Department of Ophthalmology and Shiley Eye Institute, University of California San Diego, San Diego, CA, USA; 2 Huaxi Hospital, Sichuan University, China; 3 Xijing Hospital, Xi’an, Shangxi, China; 4 Sichuan People’s Hospital, Chengdu, Sichuan, China

**Keywords:** age-related macular degeneration, oxidative stress, innate immunity, complement factor H, inflammation

## Abstract

Age-related macular degeneration (AMD) is a leading cause of vision loss affecting tens of millions of elderly worldwide. Early AMD is characterized by the appearance of soft drusen, as well as pigmentary changes in the retinal pigment epithelium (RPE). These soft, confluent drusen can progress into two forms of advanced AMD: geographic atrophy (GA, or dry AMD) or choroidal neovascularization (CNV, or wet AMD). Both forms of AMD result in a similar clinical progression in terms of loss of central vision. The exact mechanism for developing early AMD, as well as triggers responsible for progressing to advanced stage of disease, is still largely unknown. However, significant evidence exists demonstrating a complex interplay of genetic and environmental factors as causes of AMD progression. Multiple genes and/or single nucleotide polymorphisms (SNPs) have been found associated with AMD, including various genes involved in the complement pathway, lipid metabolism and extracellular matrix (ECM) remodeling. Of the known genetic contributors to disease risk, the CFH Y402H and HTRA1/ARMS polymorphisms contribute to more than 50% of the genetic risk for AMD. Environmentally, oxidative stress plays a critical role in many aging diseases including cardiovascular disease, cancer, Alzheimer’s disease and AMD. Due to the exposure to sunlight and high oxygen concentration, the oxidative stress burden is higher in the eye than other tissues, which can be further complicated by additional oxidative stressors such as smoking. Increasingly, evidence is accumulating suggesting that functional abnormalities of the innate immune system incurred via high risk genotypes may be contributing to the pathogenesis of AMD by altering the inflammatory homeostasis in the eye, specifically in the handling of oxidation products. As the eye in non-pathological instances maintains a low level of inflammation despite the presence of a relative abundance of potentially inflammatory molecules, we have previously hypothesized that the tight homeostatic control of inflammation via the innate immune system is likely critical for avoidance of disease progression. However, the presence of a multitude of potential triggers of inflammation results in a sensitive balance in which perturbations thereof would subsequently alter the inflammatory state of the retina, leading to a state of chronic inflammation and pathologic progression. In this review, we will highlight the background literature surrounding the known genetic and environmental contributors to AMD risk, as well as a discussion of the potential mechanistic interplay of these factors that lead to disease pathogenesis with particular emphasis on the delicate control of inflammatory homeostasis and the centrality of the innate immune system in this process.

## 1. Introduction

**Figure 1:**
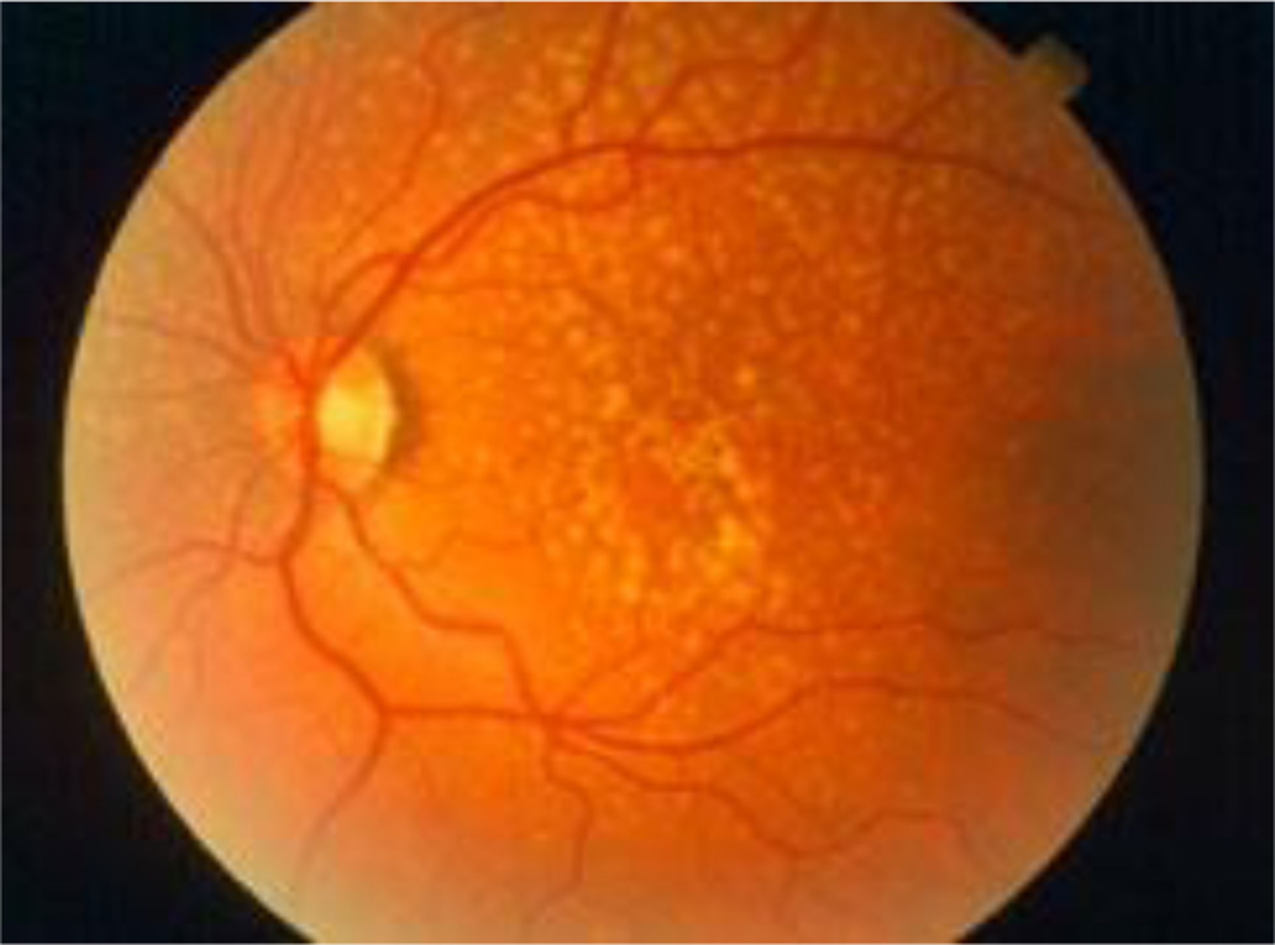
Drusen are yellow deposits under the retina, the light-sensitive tissue at the back of the eye Drusen consist of lipids and fatty protein. While not all drusen cause AMD, their presence increases a person’s risk of developing AMD. (Adapted from American Academy of Ophthalmology (7)).

**Figure 2:**
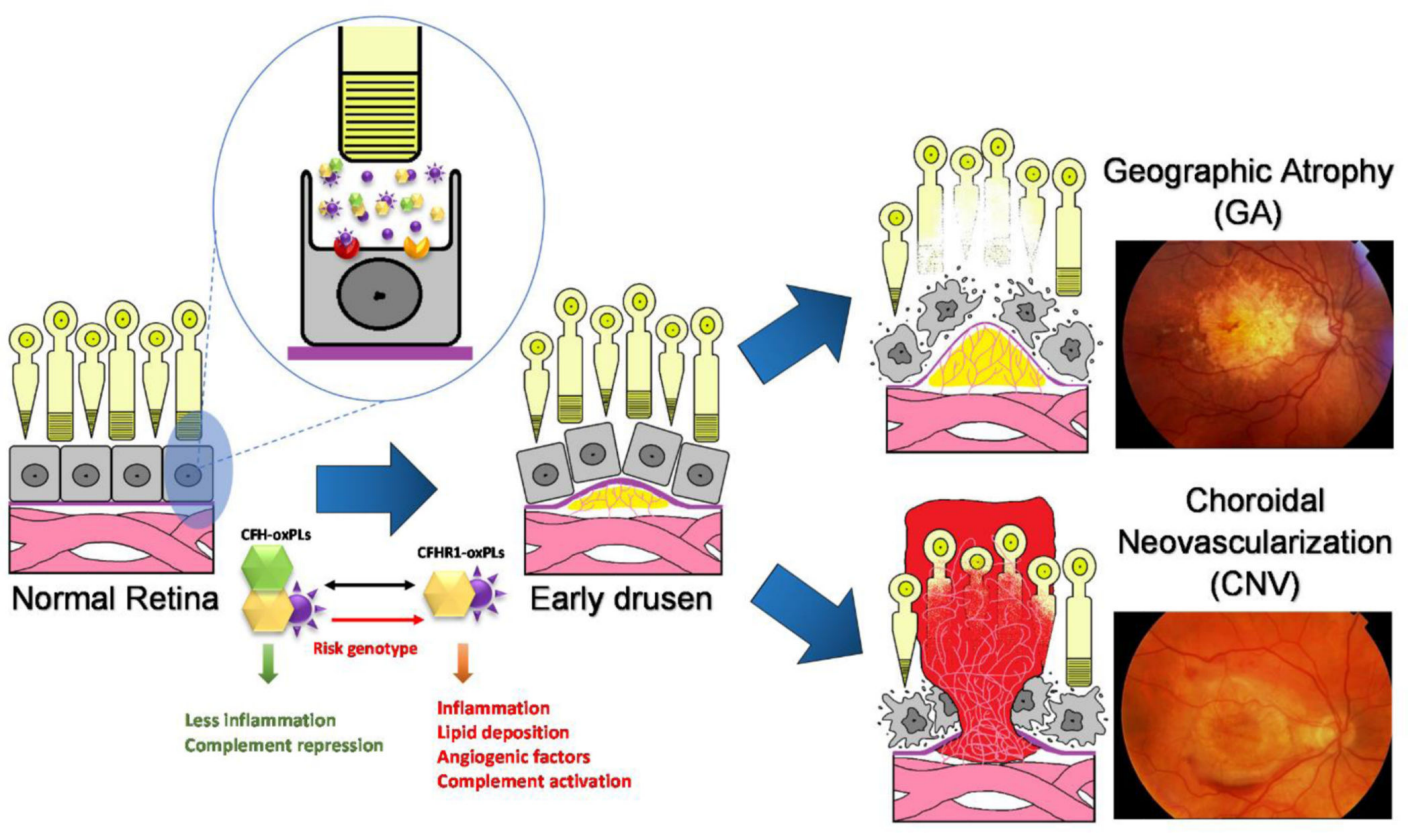
Age-related macular degeneration (AMD) is a disease that causes the progressive damage of the macula, the center of retina responsive for central and precise vision Genetic risk factors response to the environment stimulants, such as oxidative stress resulting in drusen formation, inflammation and abnormal vascular growth. The unique nature of the eye leads to an abnormal burded of both degraded lipid products and oxidative stress, leading to relatively greater burden of oxidized lipid biproducts such as oxPLs. There are two forms of advanced AMD: graphic atrophy (GA) (upper right) and choroidal neovascularization (CNV) (lower right).

Age-related macular degeneration (AMD) is a well-established disease of aging and chronic inflammation. The hallmark of the disease condition is the presence of soft drusen, yellow deposits of lipids and proteins, primarily in the area of the central region of retina called macula (Figure 1). However, the presence of confluent, soft drusen is not sufficient for the clinical diagnosis of AMD. Rather, at this early/intermediate stage of AMD tissue loss is either absent or only in its early stages and vision is usually unaffected (1). AMD progression and severity is directly correlated to the number and size of drusen. Advanced AMD occurs in 2 forms: (i) geographic atrophy (GA) of the retinal pigment epithelium (RPE) and overlying photoreceptors (also called advanced “dry” AMD), and (ii) choroidal neovascularization (CNV, also called “wet” AMD). Dry (GA) AMD is characterized by confluent areas of photoreceptor and RPE cell death, and is responsible for 10% of the legal blindness caused by AMD (2). Approximately 900,000 people in the United States are currently affected by GA with more than half of the patients occurring bilaterally (3). Wet (CNV) AMD accounts for the remaining 90% of acute blindness caused by AMD and is characterized by abnormal blood vessel growth under the macula. These new vessels are largely malformed, which leads to the improper vascular integrity causing undesirable fluid leakage within the disrupted tissue infiltrated by the unwanted vasculature (Figure 2) (4, 5).

Despite the prevalence of this disease, its etiology remains largely unknown. A growing amount of evidence has indicated that the pathogenesis and progression of AMD result from a combination of genetic risk factors and environmental insults such as smoking, UV exposure and microbial assault (6). However, as with much of the underlying physiology of AMD, the interplay between environmental factors linked to AMD and genetic variants resulting from risk-associated genetic variation remains a mystery. Efforts to understand the pathological interplay of multifactorial systems have led our lab to explore the specific interactions of risk-associated genotypes associated with complement factor H (CFH) proteins and consequences of oxidative stress, specifically those related to lipids. We found that the CFH genetic variation CFHY402H, which is association with increased AMD risk, demonstrated decreased interactivity with oxidation-modified lipids. As the association of CFH with these molecules restricted their innate inflammatory capacity, this diminished affinity disrupts of the tightly controlled inflammatory homeostasis of the eye, leading to increased inflammatory burden which is central to AMD pathogenesis (8). As the accumulation of chronic, low-level inflammation is exacerbated over time by the accumulation of oxidation products, the result is a gradual progression of disease pathology over the course of life, eventually causing tissue damage that permanently impairs the central vision, leading to blindness. Studies such as these which uncover a novel interplay between genetic and environmental disease contributors provide insight into the complex pathology involved in AMD, and are critical to furthering our understanding towards creating the next generation of approaches to AMD therapeutics. The following review investigates further the specific actors within genetic variations and environmental stressors that lead to the irreversible vision loss caused by AMD.

## 2. Genetic variants associated with AMD

**Figure 3:**
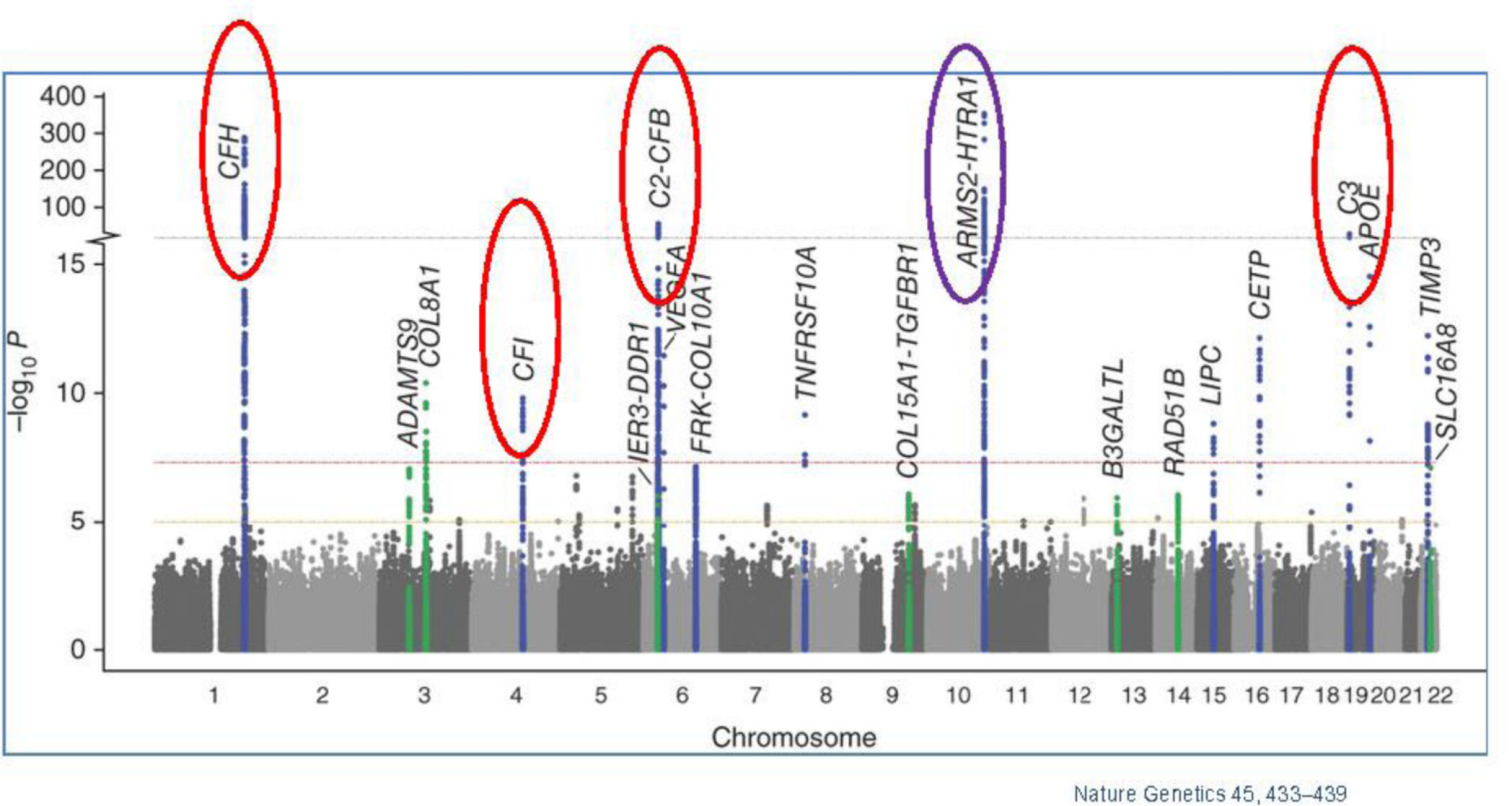
Manhattan plot showing the summary of genome-wide association results in the discovery GWAS sample The significance of association for genetic variants including single nucleotide polymorphisms (SNPs) in a genome-wide association analysis is indicated by the P values in log scale. The data set are plotted for SNPs on each chromosome with P < 5 × 10^−8^ labeled with the gene. Red circles indicate genes in complement pathway; purple circle indicates the HTRA1/ARMS2 loci. Adapted from *Nature Genetics* 45, 433–439 (9)

Genome Wide Association Studies (GWAS) have identified several genes and/or single nucleotide polymorphisms (SNPs) that associate with AMD (Figure 3). However, as mechanistic and genetic investigations of the underlying triggers of AMD pathology continue to evolve, the polygenicity and complex interplay with environmental triggers increasingly portrays AMD as a disease where variable combinations of factors converge on the same pathogenic road towards macular degeneration and vision loss. Understanding these convergence points, and the commonalities of these variable disease effectors, remains the scientific mystery likely to hold the key to understanding the true underlying mechanisms of this disease. These understanding will undoubtedly be central to developing the next generation of truly efficacious interventions. This section will provide an overview of the more substantive identified genetic contributors to AMD, as well as a brief discussion of the putative mechanistic contributions these genetic factors afford to AMD. This will be followed by a discussion of environmental contributions, with a focus on the most highly validated environmental contributor to AMD, oxidative stress. Finally, this review will discuss the potential interplay between these factors, providing additional mechanistic insight into both aspects of AMD risk. Table 1 shows the genes and SNPs that associate with AMD risk. Here we also show a Manhattan plot, which display genetic variants in genes and chromosome regions associated with AMD.

### 2.1. Genetic contributions of the complement pathway

**Figure 4:**
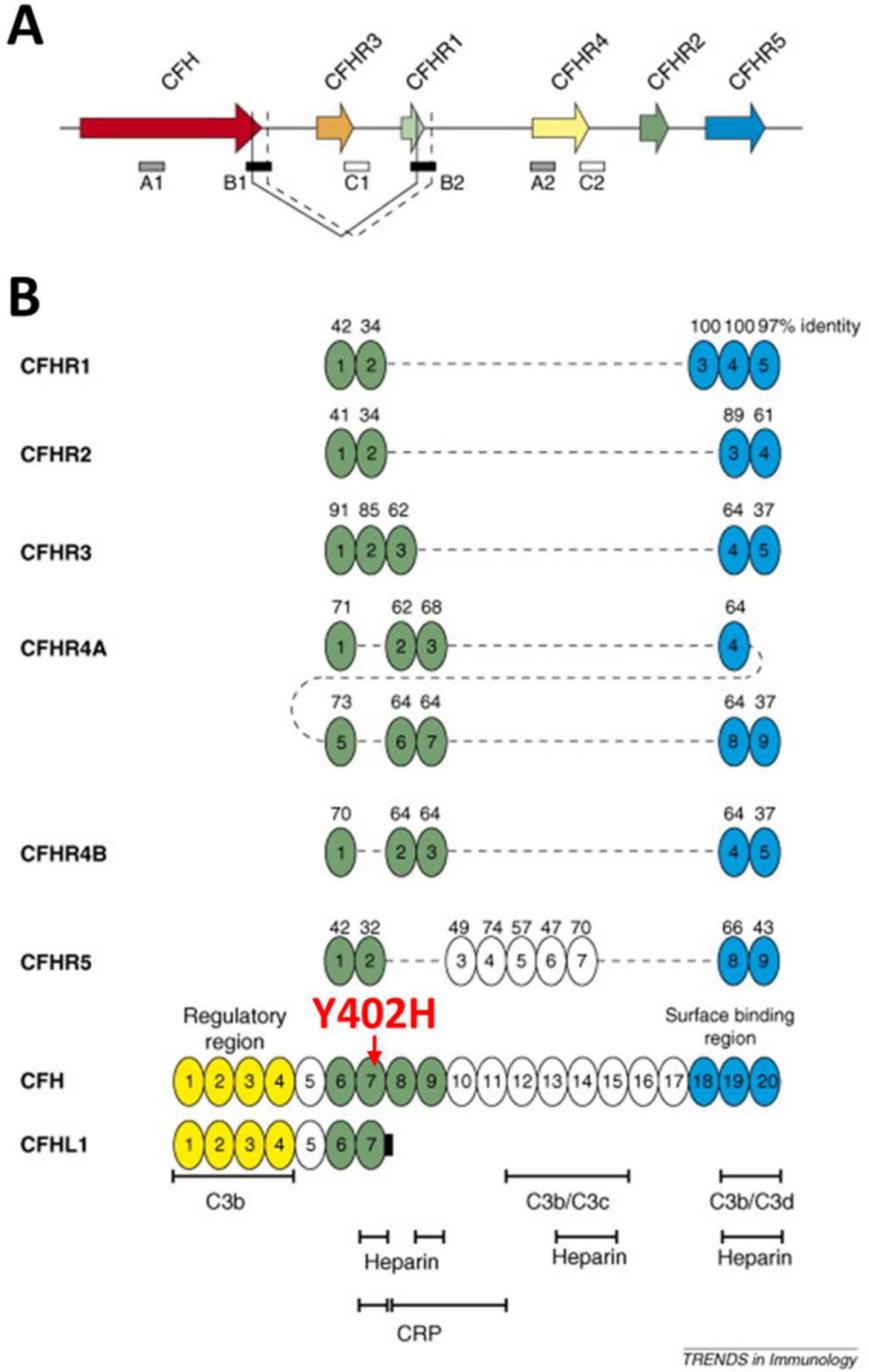
The genetic (A) and domain structures (B) of human CFH and CFHRs (adapted from (47).

The best characterized region of genetic risk associated with AMD has been repeatedly demonstrated to localize within a specific loci on chromosome one. Within this region, genetic variants associated with the alternative complement pathway in multiple forms have been identified. For example, the risk genotype of *rs1061170* that causes amino acid changes in CFH 402Y to H (CFHY402H) increases AMD risk between 2 to 4-fold for heterozygote carriers (only one allele of chromosome carries the risk variant) and 3 to 7-fold for homozygotes (both chromosome alleles carry the risk variant) (26, 27). As part of the innate defense system, the complement system is tightly regulated by many of its component factors, such as CFH and CFH related proteins (CFHRs) in modulating the alternative complement pathway. CFH, which consists of 20 short consensus repeats (SCRs), preferentially binds to host cells through glycosaminoglycans to protect host tissue from complement-mediated damage. CFHRs are composed of variation combinations of the SCRs, but all lack SCR1–4 (Figure 4 from (47)). Nevertheless, as the homology may suggest, CFH and CFHRs play similar, yet intricate, roles in regulating the complement pathway (48, 49). In the immune privileged eye, under the condition of non-infectious settings, oxidatively modified materials can be recognized “non-self” and removed by the complement system to maintain homeostasis (50, 51). Similar to naturally occurring antibodies, CFH plays an anti-inflammatory role in the eye by restricting immune activation in response to these molecules (8). The risk-associated CFHY402H mutation reduces the affinity of CFH to bind such molecules, thereby reducing its ability to maintain immune homeostasis in the eye.

**Figure 5:**
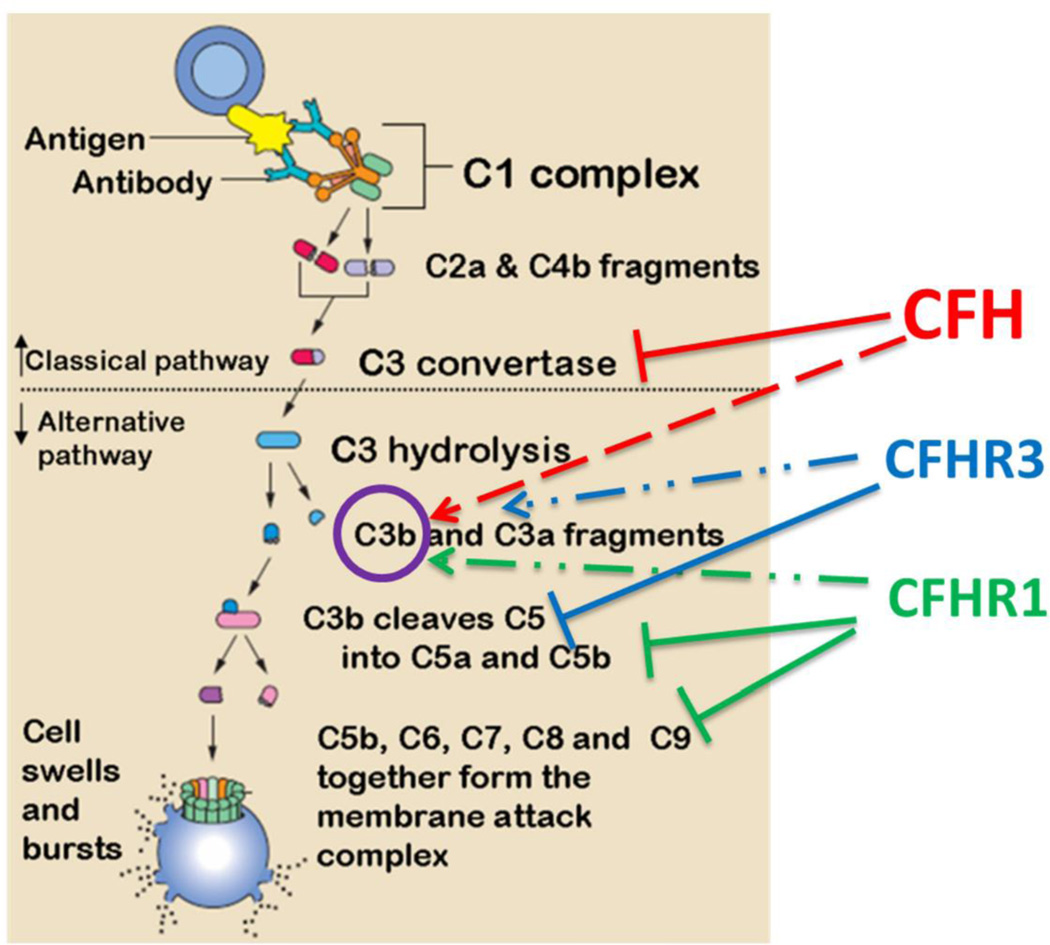
Putative role of CFH and CFHRs in regulating classical or alternative complement pathways CFH interacts with C3 convertase and also with C3b. CFHR1/3 inhibit later stages of the cascade and their deficiency of would results in a loss of complement control but enhances local regulation CFH.

While wild-type CFH has demonstrated protective benefits in AMD, expression of CFHR1 and CFHR3 demonstrate a surprising and opposite effect in their ability to increase risk of AMD occurrence (52, 53). Paradoxically, loss-of-function mutation or deletion of complement-protective CFHR1/3 (protective for AMD) is actually pathogenic in other conditions such as C3 glomerulopathy (54) and hemolytic uremic syndrome (HSU) (55). In the latter case, homozygous deletion of CFHR1/CFHR3 is strongly associated with the development of factor H auto-antibodies (DEAP HUS) (49, 55–57). The identification of CFHR1, but not CFH, in AMD drusen and Bruch’s membrane indicates an important distinction in their ability to contribute to lipid accumulation in RPE, which is likely to contribute to the inability of CFHR1/3 to maintain inflammatory homeostasis in the eye. The opposing contributions of CFH and CFHRs in conferring disease risk indicate that the suppression of complement activation by CFHRs may be mechanistically uncoupled, at least in part, from their role in promoting AMD risk (Figure 5). However, other polymorphisms in non-coding regions of CFH, or in nearby genes encoding other complement factors, also demonstrate comparably strong association with disease susceptibility. For example, two genes within the major histocompatibility complex class III region have been identified as AMD risk variants; factor B (CFB) and complement component 2 (C2) (20, 25). As genetic variations in this system can both promote and protect against disease risk, it appears that any disruption of this system can have a prominent effect on disease. In general the genetic variations in the complement pathway that contribute to disease risk putatively fall into 2 categories of disrupted control of complement-mediated immune activity: (i) alterations that lead to unchecked hyperactivation of the complement pathway, (ii) alterations that restrict the checks on protection of endogenous molecules from unwanted complement attack. In each case, either loss of “self” protection or undesirable complement activity contribute to chronic increases in low-level inflammation that can then contribute to AMD progression over time. However, the specific functions of these mutations in the specific context of environmental modifications of lipids is of particular interest. This will be discussed further in discussion of oxidative stress contributions to AMD risk.

### 2.2. Genetic risk factors involved in lipid metabolism

In addition to variations associated with the complement system, genes involved in lipid metabolism arise as the next common system associated genetic variants with links to AMD risk (58, 59). As disruption of lipid homeostasis in the eye in terms of accumulation, improper degradation, aggregated storage, etc., are hallmarks of AMD pathology, this is perhaps unsurprising. The accumulation of lipids, particularly in terms of the formation of drusen, leads to chronic inflammation in the eye, further converging the handling of lipids into the “usual suspects” of AMD progression. The cholesterol transport protein apolipoprotein E is well-established as perhaps the strongest genetic factor related to the most well-known age-related pathologies of our time; atherosclerosis and Alzheimer’s Disease (AD) (60–62). In humans, the ApoE gene is represented by 3 alleles; ApoE2, ApoE3, and ApoE4. In AD, ApoE4 is of the strongest genetic factors associated with disease risk, while ApoE2 is protective. Surprisingly, in AMD the opposite is true with ApoE2 being associated with increased risk of AMD and ApoE4 serving a protective role (63). All 3 ApoE variants can be found in the RPE and Bruch’s membrane, but while ApoE4 is protective and correlates to decreased inflammation and macrophage recruitment, ApoE2 results in increased macrophage infiltration and inflammation (64). While the complexity of this paradox is likely multifaceted, the affinity for different ApoE isoforms for amyloid-β oligomers is widely believed to play at least some role in the susceptibility to AD. As such, the opposite effect on disease risk in AMD is likely due to similar distinctions in affinity for molecules in the eye that cause inflammation. This will be discussed further in later sections.

While ApoE proteins are well established contributors to AMD, they are not the only lipid-handling proteins implicated as contributors of genetic risk to AMD. Cholesterol ester transfer protein (CETP), lipoprotein lipase (LPL) and hepatic lipase (LIPC) have all recently been reproducibly implicated as factoring into AMD risk (9, 35, 36) These proteins are central to proper handling and degradation of lipoproteins, further demonstrating the vital nature of lipid homeostasis in AMD prevention. Similarly, polymorphisms in the cholesterol exporter ABCA1 also demonstrates increased risk for AMD (36). As lipid homeostasis increasingly demonstrates a central theme in AMD pathogenesis, we will likely continue to see additional lipid-related genes with AMD influence. However, as the number of genes continues to grow, it is unlikely that any single gene will provide value as a therapeutic target. Rather, promotion of lipid homeostasis/proper lipid handling will likely prove a more rational approach to the disease.

### 2.3. Genetic variation in proteases and AMD risk

Another prominent genetic association with AMD are SNPs in or near the promoter region of the high temperature required factor A1 (HTRA1) or age-related maculopathy susceptibility protein 2 (ARMS2) genes, including *rs11200638 rs10490924, rs11200638* and *rs2293870* on chromosome 10, which are perhaps the most well-documented genetic associate with neovascular AMD (15, 18, 19). Within this genetic region lies a common disease haplotype, TAT-tagged by *rs10490924, rs11200638* and *rs2293870*, which encompasses HTRA1/ARMS2 and is significantly associated with the risk for AMD (15). HTRA1 is a serine protease that has been shown weaken extracellular matrix (ECM) (65). In addition, our previous study has characterized its ability to function as a transcriptional enhancer of VEGF expression, which has obvious implications for neovascularization. In the Bruch’s membrane, HTRA1 enzymatic activity compromises the compartmental integrity of the basal retina, which can leave the tissue susceptible to the aberrant vascularization from increased levels of VEGF. Additionally, the abnormal levels of HTRA1 found in AMD patients that causes Bruch’s membrane deterioration also leads to stimulation of inflammation of the RPE, which has also been observed *in vitro* (65).

Another protease contributor to AMD is the tissue inhibitor of metalloproteinases 3 (TIMP 3), which belongs a group of peptidases involved in degradation of the ECM. Various forms of stimulation such as oxidative stress and inflammatory cytokines can induce TIMP3 expression, and genetic variants in this gene have been associated with AMD (37–39) and the highly AMD-related autosomal dominant disorder Sorsby’s fundus dystrophy (SFD) (66). However, the role of TIMP3 in AMD is somewhat controversial as some have proposed, due to the penetrance of TIMP3 in subpopulations of patients with macular degeneration, that this particular gene may represent a specific and distinct disease subset (67).

In summary, the genetic variants at the CFH and HTRA1/ARMS2 loci contribute to major genetic risk for AMD, which lead to growing functional study aiming to elucidate the molecular mechanisms underlying AMD pathogenesis, in particular how such changes interplay with the environmental risk factors. As the number of genetic studies investigating AMD continues to grow, evidence continues to emerge indicating additional rare coding variants also involved in disease, allowing us to better pinpoint causal genes within known genetic loci. As such results are experimentally confirmed, we increasingly see the need for large sample sizes to detect new loci and genes that can inform on disease pathology (68).

## 3. Environmental contributors to AMD

AMD risk has both environmental and genetic contributions. However, as an age-related disease, it also shares common risk contributors with other chronic health ailments related more to lifestyle or medical comorbidities as opposed to environmental exposure. Conditions such as obesity, sendentary lifestyle, high cholesterol and high blood pressure have well-defined influence on promoting AMD risk (69–76). However, the ability of such factors to promote AMD progression is highly non-specific as these factors influence a variety of chronic, age associated pathologies such as atherosclerosis, diabetes, AD, etc. As such, for the sake of this review we will focus primarily on the more fundamental environmental and biological contributors even as some may be secondary to these more broadly encompassing lifestyle contributions.

### 3.1. Oxidative stress

When considering the global list of correlated factors contributing to AMD, it becomes apparent that many of these factors (i.e.: sunlight exposure, diet, smoking, vitamin D levels, etc.(75, 77, 78)) have well-documented effects on oxidative stress and its consequent inflammation. Additionally, the downstream effects of oxidative stress have a variety of disparate disease-related consequences that can influence disease progression through several avenues. As such, the following will focus to some extent on the general concept of oxidative stress and its role in other contributing aspects of AMD progression to highlight the centrality of this factor in this disease.

The contribution of oxidative stress to age-associated pathology is a common trend in many diseases such as cancer, atherosclerosis, Alzheimer’s disease and Parkinson’s disease (79–83), and is a natural consequence of many of the lifestyle-associated risk factors discussed above. The centrality of oxidative stress as a disease contributor to AMD was highlighted by the Age-Related Eye Disease Study (AREDS); a major clinical trial sponsored by the National Eye Institute which was designed to learn more about the natural history and risk factors of AMD. The study specifically evaluated the effect of high doses of vitamin C, vitamin E, beta-carotene and zinc on the progression of AMD and concluded that a variety of antioxidant agents ameliorate AMD. This study was the first to confirm in humans via rigorous assessment the importance of oxidative stress in initiation and progression of the disease (69, 76). This perspective has been supported anecdotally by several studies pinpointing smoking is the top environmental risk for developing AMD in all age and ethnic groups (74, 84–89), as well as in other ocular diseases (90).

Oxidative stress/damage in the eye can be occurred in many forms via a variety of stimuli other than smoking (91). In human eyes, local exposure sunlight combined with the high local oxygen content, which is higher than other tissues, leads to a high predisposition for oxidative burden. When combined with the systemic exposure to oxidative stressors incurred via lifestyle choices or other contributors, the relative burden of oxidative stress can rapidly become disproportionately high. While oxidative stress has been linked to a variety of specific and general modes of inflammatory promotion, oxidative stress has specific mechanistic consequences in the eye directly related to AMD pathogenesis (81, 82, 91–93).

### 3.2. Oxidative modifications in the eye

**Figure 6:**
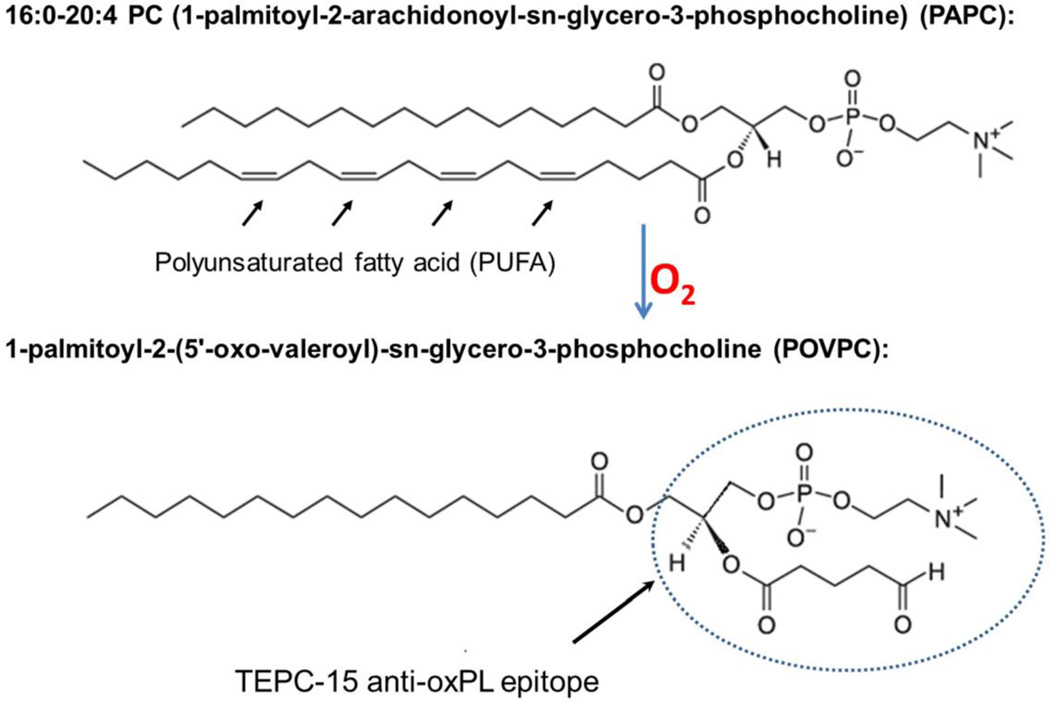
Chemical structure of PAPC, a common membrane phospholipid in the retina, and conversion to oxidatively modified POVPC, which recognized by a monoclonal antibody specific to oxPLs.

In the eye, retinal photoreceptor outer segments are constantly turned-over by retinal pigment epithelium (RPE) and enriched with polyunsaturated fatty acid (PUFA) phospholipids such as phosphatidylcholine (PC), which results in a constant supply of lipid products to be cleared and handled by the retina (94, 95). While the system is capable of maintaining such turnover, the relatively high level of oxidative burden in the eye locally and systemically results in a ready pool of lipids available for oxidative modifications. This combination can give rise to a variety of lipid modifications, particularly oxidized phospholipids (oxPLs) arising from turned over PUFAs. For example, oxidative modification of a phospholipid such as PC results in a conformational change of the head group exemplified by oxidation of 1-palmitoyl-2-arachidonoyl-*sn*-glycero-3-phosphocholine (PAPC) into 1-palmitoyl-2-(5’-oxo-valeroyl)-*sn*-glycero-3-phosphocholine (POVPC) (Figure 6). This, and similar, modifications can change otherwise ubiquitous and benign biomembrane molecule into neo-epitopes that begin to resemble that of bacterial membrane proteins, which can incite undesirable attention from immune cells (8, 96).

Oxidation of lipoproteins also produces oxysterols such as 7-ketocholesterol, 25-hydroxycholesterol, etc. When generated *in vivo*, similar to oxPLs, these are pro-inflammatory and have been shown to contribute to AD through their ability to alter cholesterol homeostasis in the brain (97), as well as atherosclerosis via activation of macrophages and stimulation of foam cell formation (98). Many oxysterols in the eye have been shown to be pro-inflammatory and cytotoxic to both photoreceptors and RPE cells (99).

Several *in vitro* and *in vivo* studies have established the association of AMD with a variety of such peroxidation-degraded lipids. During lipid peroxidation, the reactive oxygen species attack polyunsaturated fatty acids causing the breakdown of double bond that results in a variety of degraded, oxidized lipid byproducts (100). For example, enhanced lipid peroxidation necessitated in the retina for proper lipid metabolism creates several breakdown products such as malondialdehyde (MDA), malondialdehyde-acetaldehyde (MAA) (101) and POVPC. These newly modified molecules are very reactive and can facilitate interaction with a variety of matrix and structural proteins, as well as cellular membranes, to form adducts. These adducts form molecular moieties which are inherently antigenic, which can lead to improper targeting of healthy cells/molecules by the immune system causing inflammation. Consequently, studies have identified adducts of MDA (101), oxidized phosphocholine (oxPC), carboxymethyllysine (CML) (102), pentosidine (103), and carboxyethylpyrrole (CEP) (104) in AMD drusen. The fact that these oxidatively-induced modifications of autologous proteins convert ubiquitous molecules into antigenic, inflammatory stimulants is central to understanding the functional mechanism of these modifications as well indicative of the potent role of oxidative stress in AMD pathology.

In addition to its ability to modify lipid structure, oxidative stress can also lead to DNA damage, particular within mitochondria where high ROS are potent agents to mitochondrial DNA (105). Interestingly, it has been shown that DNA or RPE cells in AMD patients exhibit extensive DNA damage which leads to an inflammatory response (106). This DNA damage-related inflammation has been previously shown to not only associate with AMD, but also with aging in general (107, 108). As a result, protection of mitochondrial DNA from oxidative and other forms of damage has been proposed as a novel therapeutic strategy to slowing the progression of AMD (109).

In sum, in AMD the contribution of oxidative stress combined alterations in lipid handling caused by with the known link to genetic changes in lipid handling and inflammatory modulation indicates that inflammatory homeostasis in the eye via tight regulation of oxidized lipid products, and non-lipid byproducts, may be central to this disease.

### 3.3. Oxidative damage, inflammation, and retinal pathology

It is now clear that oxidative modifications of molecules within the eye such as those discussed above are pro-inflammatory and promote the progression of early AMD drusen to CNV or GA. The question then arises, what are the mechanisms that translate these inflammatory signals in AMD pathology? It is known that generic retinal inflammation is characteristic of a number of pathologies of the eye, and while such inflammation is undoubtedly central to AMD, chronic eye inflammation does not always lead to AMD (110). As such, identifying specific triggers and mechanisms of inflammation, as well as the participating cell types, is foundational to our understanding of this disease. We have previously discussed the prominent role of oxPLs in AMD due to the promotion of inflammation. The mechanism by which this occurs is a results of the ability of such molecules to recruit T-cells and monocytes to the subretinal tissue, where monocytes then differentiate into macrophages. These macrophages take on a strong pro-inflammatory phenotype, even sometimes resembling the morphology of foam cells readily found in atherosclerotic lesions, leading to a potent inflammatory burden within the retina (8, 111). While not directly proven to influence the transformation of early AMD to CNV, oxidized lipids have also been reported to directly affect growth, differentiation, and survival of vascular cells, which may be why some patients with wet AMD fail to respond to anti-VEGF therapy (112). In fact, studies indicate that even early forms of oxidized lipoproteins (e.g. minimally modified LDL) cause changes in gene expression (e.g. activating NFκB-like factors) of vascular cells, leading to the initiation and maintenance of an inflammatory response that could contribute to conversion of early drusen into advanced CNV (113, 114).

### 3.4. Oxidative modification and innate immunity

**Figure 7:**
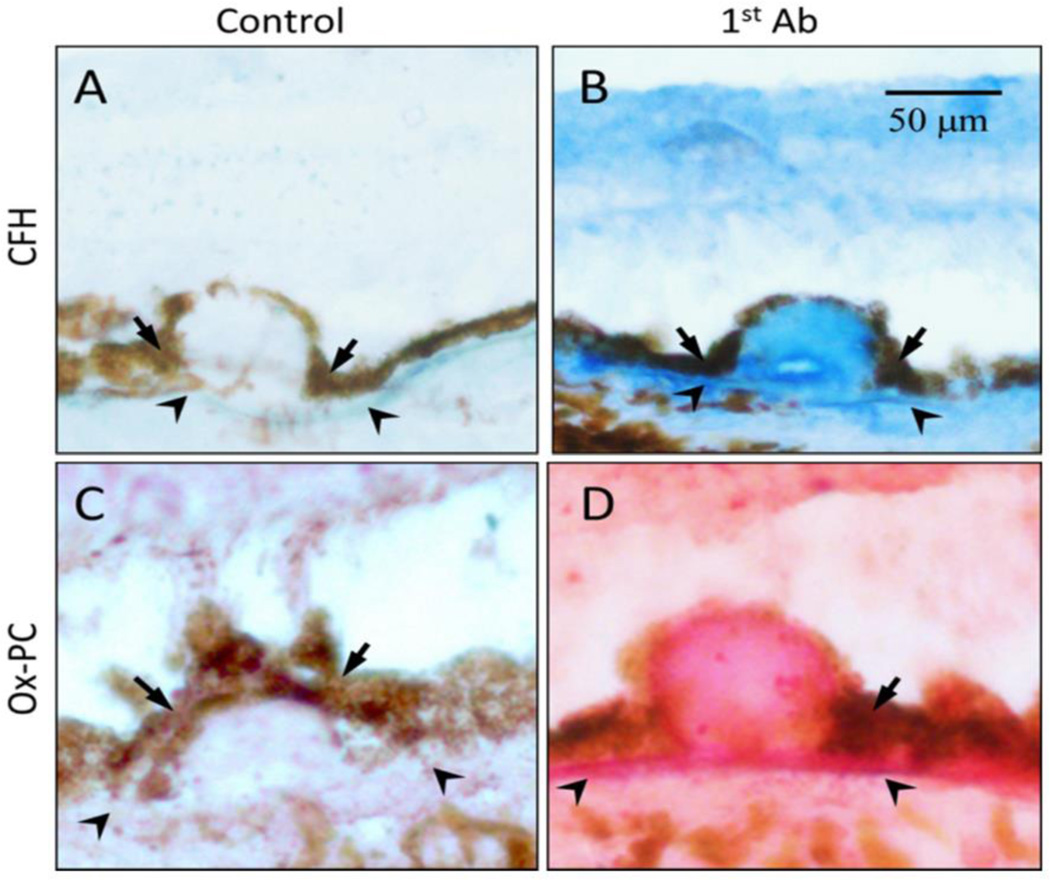
Co-localization of oxPC with CFH in human AMD lesions Immunohistochemistry of serial sections of an AMD eye stained with antibodies to CFH (Panel B, blue color) or oxPC (Panel D, pink color). Omission of 1st antibody served as a negative control (Panels A and C). Size bars: 50 microns. (8)

Despite the overwhelming evidence that complement pathway gene polymorphisms strongly associate with AMD disease risk, the mechanism and pathways through which oxidative stress affect AMD are not yet clear. Our lab has recently identified a novel mechanistic interplay between the genetic variations in CFH associated with disease risk and oxidative modifications of lipids. Although eye is an immune privileged organ, CFH protein have been observed in retina and AMD drusen (Figure 7) indicating its role that may not directly involve in regulation complement activation. Wild-type CFH demonstrates a substantially increased affinity for oxidatively modified lipids such as oxPLs and malondialdehyde (MDA) compared to native, un-oxidized lipids (8, 115, 116). As these oxidized lipids have inflammatory capacity, the interaction of CFH acts to restrict this inflammatory potential in a similar fashion to naturally occurring antibodies of the innate immune system (115). Risk-associated genetic variants either decrease the affinity of CFH for these oxidation products, or displace CFH with CFH- related proteins that lack the same anti-inflammatory capacity. As such, the oxPLs burden has a higher propensity for RPE interaction leading to inflammation and drusen formation in these patients.

Innate immune system protect host from lethal pathogenic microbial assults before adaptive innunity kicks in by recognizing pathogen-associated molecular patterns (PAMPs). Previously, studies have made an interesting finding that in addition to defending the host from microbial infection, natural antibodies, which are a branch of innate immunity, can shield host from oxidative damages, thereby preventing un-wanted inflammation, particularly in response to oxidation-specific neo-epitopes (96). These antibodies prevent the immune system from inappropriately reacting to self-proteins as pathogens, which is normally an interaction restricted to PAMPs on microbial surface or debris. Oxidized lipid products can sometimes mimic PAMPs, requiring intrinsic mechanisms to control for attack of these non-pathogenic structures. For example, mouse natural antibody TEPC-15 (aka T15), which protects neonatal animals from fetal pneumococcal infection before adaptive immune response, recognizes and interacts with phosphocholine (PC) n-linked on the cell wall of certain bacteria such as *S. pneumococci*. However, this anti-PC antibody does not recognize the PC structure o-linked to mammalian cell membrane phospholipids. After oxidative modification of phospholipids as illustrated in the Figure 6, the conformational change will present modified PC head-group to the host immune system as a neo-antigen resembling those on bacterial cell walls. Therefore, these modified cellular and molecular structures from endogenous oxidative damage are also called damage associated molecular patterns (DAMPs). Interestingly, after oxidative modification, when phospholipids on cell membrane become oxPLs, the same PC epitope can be recognized and interacted by T15. As such, T15 is able to interact with endogenous oxPC on cell membranes or lipoproteins and maintain homeostasis by turnover of unhealthy cells and macromolecules such as to remove apoptotic cells and oxLDL (96, 115).

### 3.5. Immune cells, inflammation and AMD progression

Even though the eye is an immune privileged organ, the presence of resident immunocompetent cells (e.g. microglia), and humoral factors such as cytokines, adhesion molecules, auto-antibodies, and acute phase proteins lead to a somewhat self-contained immune system that can play important roles in determining the inflammatory status of the retina. However, understanding the specific cellular contributors and signals unique to AMD is important in the understanding of this disease. Unsurprisingly, a panel of pro-inflammatory cytokines and chemokines, including IL-1, IL-6, IL-8, TNF, INF-γ, MCP-1, have been shown to accelerate the AMD progression (117). In addition, tissue factors that have pro-angiogenic activity, such as VEGF A-E, platelet-derived growth factor (PDGF), placental growth factor (PlGF), hepatocyte growth factor (HGF), and fibroblast growth factor-2 (FGF-2), are also involved in CNV formation (118).

Recently it was shown that CFHY402H patients expressed significantly greater levels of INFγ-inducible protein-10 (IP-10) and eotaxin, perhaps indicating such factors may be useful as vitreal biomarkers of early AMD (119). As this was uniquely identified in this specific genetic background, future studies to assess the incidence of elevation of these markers in non CFHY402H AMD patients will be of great interest. Similarly, it has also been shown in CFHY402H patients that macrophage infiltration is likely stimulated by elevated levels of vitreal granulocyte macrophage colony-stimulating factor (GM-CSF), leading to detection of choroidal macrophages in the postmortem human eye (120). The role of these macrophages is yet unclear, as these cells can accumulate in sites of damage or disease without contributing to disease progression, in some cases even serving a beneficial purpose. However, a recent study demonstrated a large population of CD163 positive cells in the retina of wet and dry AMD postmortem human samples (121). As CD163 is associated with potently inflammatory immune populations, it is likely that macrophages present in the retina of AMD patients are not serving any putative disease restricting purpose. Regardless, these specific, localized inflammatory events continue to provide insight into the unique players involved in promotion of inflammatory disease within an immune-privileged tissue. As such, further characterization and mechanistic elucidation could identify novel therapeutic avenues by which disruption of disease-associated cellular recruitment, or inflammatory status of infiltrating cellular populations, could slow the progression of vision loss.

The infiltration of macrophages from the peripheral circulation is a unique and interesting component of AMD pathology. The role of these macrophages, as well as the specific functions of the pro-inflammatory (M1) and inflammation suppressing (M2) phenotypes in regulating AMD pathology, is currently an important topic of active investigation (111). M1 macrophages in the retina contribute to the elevated levels of inflammatory cytokine/chemokines known to be associated with AMD such as IL1, IL6, IL8, and the production of VEGF. Conversely, while M2 macrophages do not express the inflammatory milieu characteristic of M1, they do produce pro-angiogenic factors, such as basic fibroblast growth factor (FGF-2), insulin-like growth factor-1 (IGF-1), and placental growth factor (PGF) (110). As a result, while M2 macrophages are largely considered beneficial to disease, this pro-angiogenic function could still contribute to wet AMD progression despite expression of anti-inflammatory cytokines such as IL-10 and IL-18 that have shown to have protective effect in regard to AMD risk (122). Additionally, while M1 macrophages are commonly considered pathogenic, their expression of the cytokine IL-18 has been shown to combat the neovascular effects of VEGF via attenuation of CNV in rodent models, as well as in nonhuman primate models of wet AMD, thereby indicating a potential therapeutic role for M1 macrophages in this pathology (123, 124)). Future work should elaborate on the intricacy of macrophage function in these diseases, and the specific contributions of M1 vs. M2 phenotypes.

## 4. Immune response in GA and CNV

The commonalities between wet and dry AMD are numerous in their early stages. The strong association of complement pathway genes with AMD susceptibility (125) and the presence of complement proteins in drusen indicate that AMD is likely, at least in part, a chronic inflammatory disease involving abnormal regulation of complement and immune system (25, 126–130),(131). Proteomic studies have found that many oxidatively modified proteins and lipids as well as immunoglobulins in AMD drusen (91, 131, 132). Such oxidative materials including oxPLs are strong stimulants to the immune system resulting in promoting inflammation and activating the complement cascade in RPE cells. Thus in early and intermediate stages of AMD, the immune responses to oxidative damage play important roles in RPE apoptosis and lipids accumulation leading to drusen formation.

Despite the early hallmark pathology for both forms of wet and dry AMD to be commonly held, a divergence point seems defined by the degradation of the Bruch’s membrane and parallel development of malformed vasculature infiltration. The clearest differentiating contributor to this pathological distinction is the role of VEGF. However, VEGF levels are most likely a secondary consequence of the pathological mechanisms at play as opposed to directly related to disease progression due to some form of unwanted, and untriggered, overexpression. The SNPs in or near the promoter region of the high temperature required factor A1 (HTRA1) or age-related maculopathy susceptibility protein 2 (ARMS2) genes on chromosome 10 include *rs11200638 rs10490924, rs11200638* and *rs2293870* are the strongest genetic contributor to wet AMD and these result in increased production of VEGF in the retina. (19, 65). In addition to its characterized ability to function as a transcriptional enhancer of VEGF expression, which has obvious implications for CNV, HTRA1 is also a serine protease that has been shown weaken ECM (65, 133). In the Bruch’s membrane, this enzymatic activity compromises the compartmental integrity of the basal retina, which can leave the tissue susceptible to the aberrant vascularization that results from increased levels of VEGF. Additionally, the abnormal levels of HTRA1 found in AMD patients that causes Bruch’s membrane deterioration also leads to stimulation of inflammation of the RPE, which has also been observed *in vitro* (65). While it has not been tested, TIMP3 may also contribute to CNV pathogenesis in a similar fashion.

Although the specific role of the macrophage lineage cells at different stages of AMD is still controversial, these components of innate immune system play an important role in neovascular AMD (134, 135). The macrophages recruited in the retina are from two sources. The first one belongs to microglia, which are bone marrow-derived resident macrophages recruited to neural tissue during retinal development. They provide immune surveillance in the inner retina and have been associated with AMD (136, 137). The second group is from circulating monocytes that can be recruited from the blood vessels to sites of inflammation by specific chemokines and cytokines, which are stimulated by oxidative in the retina and RPE (138, 139). Regardless of the source, macrophages can undergo further differentiation depending under the oxidative microenvironment and eventually perform their effector functions (140, 141). For example the presence of extracellular nitric oxide synthase (iNOS) is associated with macrophage recruitment to Bruck’s membrane and alteration in the immunophenotype of resident choroidal macrophages (135, 142). In the presence of interferon-gamma (IFN-γ), macrophages are activated as proinflammatory M1 macrophages, which produce tumor necrosis factor alpha (TNF-α) and interleukin-12 (IL-12) and are associated with tissue damage (143).

## 5. Conclusions

In the past decade, there has been an unprecedented increase in the understanding of genetic and environmental contributions to AMD. These novel insights have begun to frame a general interplay of extrinsic and intrinsic factors that contribute to important homeostatic aspects of the eye such as inflammation and lipid processing, but the precise mechanisms contributing to these pathologic disruptions are still poorly understood. By continuing to investigate the functional consequence of genetic variants contributing to AMD such as those found in 1q31–32 and 10q26, which represent genes from the complement system and serine protease family respectively, we can continue to identify biological functions/systems that are repeatedly influenced by identified genetic variants. In turn, we can continue to perform hypothesis-driven research into the potential mechanisms between specific genetic variations and known environmental contributors to disease risk to uncover novel points of intervention within these systems that can preserve vision and improve patient outcomes. While genetic factors are well-characterized as potent contributors to AMD pathogenesis, the disproportionate emphasis on such studies in AMD research is unlikely to catalyze future treatments in an efficient manner. Genetic variations are determined at birth, and while the contribution to risk is quite real, the person-to-person likelihood of developing AMD as a result of carrying these genetic risk factors is often quite minimal, and even then only later in life. As such, these findings are unlikely to provide value proportionate to the effort and funding invested in their acquisition unless subsequently applied to hypothesis-driven studies aimed at elucidating the interplay with environmental or cooperative disease triggers. The in-depth understanding of the interplay between genetic and environmental factors leading to AMD is likely the key to discovering novel therapeutic interventions capable of salvaging vision in these patients.

## Figures and Tables

**Table 1:** Genes/SNPs with published AMD associations.

*Genes*	Variants (SNP)	Full Name	Function	Position	Odds Ratios	References
*ABCA4*	*rs1800553*, *rs1800555*	ATP-binding cassette, sub-family A (ABC1), member 4	Photoreceptor specific expression; transport of N-retinylidene-PE across the outer segment disc membrane	1p22	N.A.	(10–12)
*APOE*	*rs429358, rs7412*	apolipoprotein E	lipid and cholesterol transport	19q13	ε2 OR_homo_ = 1.046; ε*4* OR_homo_ = 0.847 (Thakkinstian); ε4 OR_homo_ = 0.35–0.53, 0.847 (separate report-Francis)	(13, 14)
ARMS2 /LOC387715*	*rs10490924, in/del* *(372_815delins54)*	age-related maculopathy susceptibility 2	no known function; protein localized to mitochondrial outer membrane	10q26	OR_homo_ = 8.59 (rs10490924)	(12, 15–17)
HTRA1*	*rs11200638*	human High Temperature Requirement A1	trypsin-like serine protease	10q26	OR_homo_ = 6.92, 7.46	(6, 18, 19)
*C2/CFB*	*rs9332739 (c2)*, *rs4151667(CFB)*, *rs641153 (CFB)*	complement 2/ complement factor B	regulation of complement activation	6p21	OR_hetero_ = 0.21–0.45	(6, 20)
*C3*	*rs2230199*, *rs1047286*	complement 3	Innate immunity (alternative complement pathway activator, classical pathway component)	19p13	OR_homo_ = 1.93–3.26	(21–23)
*CETP*	*rs1864163*	Cholesteryl ester transfer protein	Transfer cholesteryl esters between lipoproteins	16q21	OR_homo_ = 1.2	(24)
CFH*	*rs1061170*	complement factor H	inhibitor of alternative complement pathway	1q32	OR_homo_ = 6.35	(25–28)
*CFHR1/CFHR3*	*84K bp deletion*	complement factor H-related 1, 3	exact function unknown, possible overlapping function with *CFH*	1q31-q32	OR_homo_ = 0.29	(29, 30)
*CFI*	*rs4698775*	Complement factor I	regulation of complement activation	4q25	OR_homo_ = 1.1	(31)
*CX3CR1*	*rs3732378*	chemokine (C-X3-C motif) receptor 1	Inflammatory (chemokine receptor)	3p21	OR_homo_ = 1.98–2.70	(32, 33)
*ERCC6*	*rs3793784*	excision-repain cross-complementing, group6	DNA repair	10q11	OR_homo_ = 1.6	(34)
*LIPC*	*rs920915*	Hepatic lipase	Lipid metabolism	15q21-23	OR_hetero_ = 1.1	(24, 35, 36)
*TIMP3*	*rs9621532*	Tissue inhibitor of metalloproteinase	Complexes with inactive metalloproteinase	22q12	OR_homo_ = 1.31–1.91	(37–39)
*TLR3*	rs3775291	toll-like receptor 3	Innate immunity (encodes protein to recognize viral dsRNA)	4q35	OR_homo_ = 0.44–0.61	(40, 41)
*TLR4*	*rs4986790*	toll-like receptor 4	Innate immunity (encodes bacterial endotoxin receptor)	9q32-q33	OR_hetero_ = 2.65	32 (42, 43)
*VEGFA*	*rs833069*, *rs1413711*	vascular endothelial growth factor A	Angiogenic and vasculogenic growth factor	6p12	OR_homo_ = 5.29 (rs833069), OR_homo_ = 2.40 (rs1413711)	(44–46)

* OR_homo_ indicates odds ratio from homozygous; OR_hetero_ indicates odds ratio of heterozygous.

## References

[1] Jager RD, Mieler WF, Miller JW (2008). Age-related macular degeneration. The New England journal of medicine.

[2] Sunness JS (1999). The natural history of geographic atrophy, the advanced atrophic form of age-related macular degeneration. Molecular vision.

[3] Ying GS, Maguire MG, and Complications of Age-related Macular Degeneration Prevention Trial Research, G (2011). Development of a risk score for geographic atrophy in complications of the age-related macular degeneration prevention trial. Ophthalmology.

[4] Sarks JP, Sarks SH, Killingsworth MC (1988). Evolution of geographic atrophy of the retinal pigment epithelium. Eye (Lond).

[5] Ferris FL, Fine SL, Hyman L (1984). Age-related macular degeneration and blindness due to neovascular maculopathy. Arch Ophthalmol.

[6] Chen Y, Zeng J, Zhao C, Wang K, Trood E, Buehler J, Weed M, Kasuga D, Bernstein PS, Hughes G (2011). Assessing susceptibility to age-related macular degeneration with genetic markers and environmental factors. Arch Ophthalmol.

[7] Ophthalmology.

[8] Shaw PX, Zhang L, Zhang M, Du H, Zhao L, Lee C, Grob S, Lim SL, Hughes G, Lee J (2012). Complement factor H genotypes impact risk of age-related macular degeneration by interaction with oxidized phospholipids. Proceedings of the National Academy of Sciences of the United States of America.

[9] Fritsche LG, Chen W, Schu M, Yaspan BL, Yu Y, Thorleifsson G, Zack DJ, Arakawa S, Cipriani V, Ripke S (2013). Seven new loci associated with age-related macular degeneration. Nat Genet.

[10] Beharry S, Zhong M, Molday RS (2004). N-retinylidene-phosphatidylethanolamine is the preferred retinoid substrate for the photoreceptor-specific ABC transporter ABCA4 (ABCR). J Biol Chem.

[11] Allikmets R (2000). Further evidence for an association of ABCR alleles with age-related macular degeneration. The International ABCR Screening Consortium. Am J Hum Genet.

[12] Katta S, Kaur I, Chakrabarti S (2009). The molecular genetic basis of age-related macular degeneration: an overview. J Genet.

[13] Thakkinstian A, Bowe S, McEvoy M, Smith W, Attia J (2006). Association between apolipoprotein E polymorphisms and age-related macular degeneration: A HuGE review and meta-analysis. American journal of epidemiology.

[14] McKay GJ, Silvestri G, Chakravarthy U, Dasari S, Fritsche LG, Weber BH, Keilhauer CN, Klein ML, Francis PJ, Klaver CC (2011). Variations in apolipoprotein E frequency with age in a pooled analysis of a large group of older people. American journal of epidemiology.

[15] Fritsche LG, Loenhardt T, Janssen A, Fisher SA, Rivera A, Keilhauer CN, Weber BH (2008). Age-related macular degeneration is associated with an unstable ARMS2 (LOC387715) mRNA. Nat Genet.

[16] Ross RJ, Bojanowski CM, Wang JJ, Chew EY, Rochtchina E, Ferris FL, Mitchell P, Chan CC, Tuo J (2007). The LOC387715 polymorphism and age-related macular degeneration: replication in three case-control samples. Invest Ophthalmol Vis Sci.

[17] Jakobsdottir J, Conley YP, Weeks DE, Mah TS, Ferrell RE, Gorin MB (2005). Susceptibility genes for age-related maculopathy on chromosome 10q26. Am J Hum Genet.

[18] Dewan A, Liu M, Hartman S, Zhang SS, Liu DT, Zhao C, Tam PO, Chan WM, Lam DS, Snyder M (2006). HTRA1 promoter polymorphism in wet age-related macular degeneration. Science.

[19] Yang Z, Camp NJ, Sun H, Tong Z, Gibbs D, Cameron DJ, Chen H, Zhao Y, Pearson E, Li X (2006). A variant of the HTRA1 gene increases susceptibility to age-related macular degeneration. Science.

[20] Gold B, Merriam JE, Zernant J, Hancox LS, Taiber AJ, Gehrs K, Cramer K, Neel J, Bergeron J, Barile GR (2006). Variation in factor B (BF) and complement component 2 (C2) genes is associated with age-related macular degeneration. Nat Genet.

[21] Yates JR, Sepp T, Matharu BK, Khan JC, Thurlby DA, Shahid H, Clayton DG, Hayward C, Morgan J, Wright AF (2007). Complement C3 variant and the risk of age-related macular degeneration. The New England journal of medicine.

[22] Maller JB, Fagerness JA, Reynolds RC, Neale BM, Daly MJ, Seddon JM (2007). Variation in complement factor 3 is associated with risk of age-related macular degeneration. Nat Genet.

[23] Francis PJ, Hamon SC, Ott J, Weleber RG, Klein ML (2009). Polymorphisms in C2, CFB and C3 are associated with progression to advanced age related macular degeneration associated with visual loss. Journal of medical genetics.

[24] Chen W, Stambolian D, Edwards AO, Branham KE, Othman M, Jakobsdottir J, Tosakulwong N, Pericak-Vance MA, Campochiaro PA, Klein ML (2010). Genetic variants near TIMP3 and high-density lipoprotein-associated loci influence susceptibility to age-related macular degeneration. Proc Natl Acad Sci U S A.

[25] Hageman GS, Anderson DH, Johnson LV, Hancox LS, Taiber AJ, Hardisty LI, Hageman JL, Stockman HA, Borchardt JD, Gehrs KM (2005). A common haplotype in the complement regulatory gene factor H (HF1/CFH) predisposes individuals to age-related macular degeneration. Proc Natl Acad Sci U S A.

[26] Wegscheider BJ, Weger M, Renner W, Steinbrugger I, Marz W, Mossbock G, Temmel W, El-Shabrawi Y, Schmut O, Jahrbacher R (2007). Association of complement factor H Y402H gene polymorphism with different subtypes of exudative age-related macular degeneration. Ophthalmology.

[27] Klein RJ, Zeiss C, Chew EY, Tsai JY, Sackler RS, Haynes C, Henning AK, SanGiovanni JP, Mane SM, Mayne ST (2005). Complement factor H polymorphism in age-related macular degeneration. Science.

[28] Thakkinstian A, Han P, McEvoy M, Smith W, Hoh J, Magnusson K, Zhang K, Attia J (2006). Systematic review and meta-analysis of the association between complement factor H Y402H polymorphisms and age-related macular degeneration. Hum Mol Genet.

[29] Spencer KL, Hauser MA, Olson LM, Schmidt S, Scott WK, Gallins P, Agarwal A, Postel EA, Pericak-Vance MA, Haines JL (2008). Deletion of CFHR3 and CFHR1 genes in age-related macular degeneration. Hum Mol Genet.

[30] Raychaudhuri S, Ripke S, Li M, Neale BM, Fagerness J, Reynolds R, Sobrin L, Swaroop A, Abecasis G, Seddon JM (2010). Associations of CFHR1-CFHR3 deletion and a CFH SNP to age-related macular degeneration are not independent. Nature genetics.

[31] Fagerness JA, Maller JB, Neale BM, Reynolds RC, Daly MJ, Seddon JM (2008). Variation near complement factor I is associated with risk of advanced AMD. Eur J Hum Genet.

[32] Combadi xE, re C, Feumi C, Raoul W, Keller N, Rod xE, ro M, xE CX3CR1-dependent subretinal microglia cell accumulation is associated with cardinal features of age-related macular degeneration. The Journal of Clinical Investigation.

[33] Tuo J, Smith BC, Bojanowski CM, Meleth AD, Gery I, Csaky KG, Chew EY, Chan C-C (2004). The involvement of sequence variation and expression of CX3CR1 in the pathogenesis of age-related macular degeneration. The FASEB Journal.

[34] Tuo J, Ning B, Bojanowski CM, Lin Z-N, Ross RJ, Reed GF, Shen D, Jiao X, Zhou M, Chew EY (2006). Synergic effect of polymorphisms in ERCC6 5′ flanking region and complement factor H on age-related macular degeneration predisposition. Proceedings of the National Academy of Sciences.

[35] Neale BM, Fagerness J, Reynolds R, Sobrin L, Parker M, Raychaudhuri S, Tan PL, Oh EC, Merriam JE, Souied E (2010). Genome-wide association study of advanced age-related macular degeneration identifies a role of the hepatic lipase gene (LIPC). Proc Natl Acad Sci U S A.

[36] Yu Y, Reynolds R, Fagerness J, Rosner B, Daly MJ, Seddon JM (2011). Association of variants in the LIPC and ABCA1 genes with intermediate and large drusen and advanced age-related macular degeneration. Invest Ophthalmol Vis Sci.

[37] Ardeljan D, Meyerle CB, Agron E, Wang JJ, Mitchell P, Chew EY, Zhao J, Maminishkis A, Chan CC, Tuo J (2013). Influence of TIMP3/SYN3 polymorphisms on the phenotypic presentation of age-related macular degeneration. Eur J Hum Genet.

[38] Chen W, Stambolian D, Edwards AO, Branham KE, Othman M, Jakobsdottir J, Tosakulwong N, Pericak-Vance MA, Campochiaro PA, Klein ML (2010). Genetic variants near TIMP3 and high-density lipoprotein-associated loci influence susceptibility to age-related macular degeneration. Proc Natl Acad Sci U S A.

[39] Kaur I, Rathi S, Chakrabarti S (2010). Variations in TIMP3 are associated with age-related macular degeneration. Proceedings of the National Academy of Sciences.

[40] Yang Z, Stratton C, Francis PJ, Kleinman ME, Tan PL, Gibbs D, Tong Z, Chen H, Constantine R, Yang X (2008). Toll-like receptor 3 and geographic atrophy in age-related macular degeneration. New England Journal of Medicine.

[41] Edwards AO, Chen D, Fridley BL, James KM, Wu Y, Abecasis G, Swaroop A, Othman M, Branham K, Iyengar SK (2008). Toll-like receptor polymorphisms and age-related macular degeneration. Investigative ophthalmology & visual science.

[42] Ferwerda B, McCall MB, Alonso S, Giamarellos-Bourboulis EJ, Mouktaroudi M, Izagirre N, Syafruddin D, Kibiki G, Cristea T, Hijmans A (2007). TLR4 polymorphisms, infectious diseases, and evolutionary pressure during migration of modern humans. Proceedings of the National Academy of Sciences.

[43] Zareparsi S, Buraczynska M, Branham KE, Shah S, Eng D, Li M, Pawar H, Yashar BM, Moroi SE, Lichter PR (2005). Toll-like receptor 4 variant D299G is associated with susceptibility to age-related macular degeneration. Human molecular genetics.

[44] Galan A, Ferlin A, Caretti L, Buson G, Sato G, Frigo AC, Foresta C (2010). Association of age-related macular degeneration with polymorphisms in vascular endothelial growth factor and its receptor. Ophthalmology.

[45] Fang AM, Lee AY, Kulkarni M, Osborn MP, Brantley MA (2009). Polymorphisms in the VEGFA and VEGFR-2 genes and neovascular age-related macular degeneration.

[46] Churchill AJ, Carter JG, Ramsden C, Turner SJ, Yeung A, Brenchley PE, Ray DW (2008). VEGF polymorphisms are associated with severity of diabetic retinopathy. Investigative ophthalmology & visual science.

[47] Jozsi M, Zipfel PF (2008). Factor H family proteins and human diseases. Trends in immunology.

[48] Fritsche LG, Lauer N, Hartmann A, Stippa S, Keilhauer CN, Oppermann M, Pandey MK, Kohl J, Zipfel PF, Weber BH (2010). An imbalance of human complement regulatory proteins CFHR1, CFHR3 and factor H influences risk for age-related macular degeneration (AMD). Hum Mol Genet.

[49] Zipfel PF, Edey M, Heinen S, Jozsi M, Richter H, Misselwitz J, Hoppe B, Routledge D, Strain L, Hughes AE (2007). Deletion of complement factor H-related genes CFHR1 and CFHR3 is associated with atypical hemolytic uremic syndrome. PLoS Genet.

[50] Zipfel PF, Skerka C (2009). Complement regulators and inhibitory proteins. Nature reviews. Immunology.

[51] Ricklin D, Hajishengallis G, Yang K, Lambris JD (2010). Complement: a key system for immune surveillance and homeostasis. Nature immunology.

[52] Hughes AE, Orr N, Esfandiary H, Diaz-Torres M, Goodship T, Chakravarthy U (2006). A common CFH haplotype, with deletion of CFHR1 and CFHR3, is associated with lower risk of age-related macular degeneration. Nat Genet.

[53] Zhao J, Wu H, Khosravi M, Cui H, Qian X, Kelly JA, Kaufman KM, Langefeld CD, Williams AH, Comeau ME (2011). Association of genetic variants in complement factor H and factor H-related genes with systemic lupus erythematosus susceptibility. PLoS Genet.

[54] Tortajada A, Yebenes H, Abarrategui-Garrido C, Anter J, Garcia-Fernandez JM, Martinez-Barricarte R, Alba-Dominguez M, Malik TH, Bedoya R, Cabrera Perez R (2013). C3 glomerulopathy-associated CFHR1 mutation alters FHR oligomerization and complement regulation. J Clin Invest.

[55] Zipfel PF, Mache C, Muller D, Licht C, Wigger M, Skerka C (2010). DEAP-HUS: deficiency of CFHR plasma proteins and autoantibody-positive form of hemolytic uremic syndrome. Pediatric nephrology.

[56] Jozsi M, Licht C, Strobel S, Zipfel SL, Richter H, Heinen S, Zipfel PF, Skerka C (2008). Factor H autoantibodies in atypical hemolytic uremic syndrome correlate with CFHR1/CFHR3 deficiency. Blood.

[57] Dragon-Durey MA, Blanc C, Marliot F, Loirat C, Blouin J, Sautes-Fridman C, Fridman WH, Fremeaux-Bacchi V (2009). The high frequency of complement factor H related CFHR1 gene deletion is restricted to specific subgroups of patients with atypical haemolytic uraemic syndrome. Journal of medical genetics.

[58] Kishan AU, Modjtahedi BS, Martins EN, Modjtahedi SP, Morse LS (2011). Lipids and age-related macular degeneration. Surv Ophthalmol.

[59] Klein R, Myers CE, Buitendijk GH, Rochtchina E, Gao X, de Jong PT, Sivakumaran TA, Burlutsky G, McKean-Cowdin R, Hofman A (2014). Lipids, lipid genes, and incident age-related macular degeneration: the three continent age-related macular degeneration consortium. American journal of ophthalmology.

[60] Martiskainen H, Haapasalo A, Kurkinen KM, Pihlajamaki J, Soininen H, Hiltunen M (2013). Targeting ApoE4/ApoE receptor LRP1 in Alzheimer’s disease. Expert Opin Ther Targets.

[61] Liu CC, Kanekiyo T, Xu H, Bu G (2013). Apolipoprotein E and Alzheimer disease: risk, mechanisms and therapy. Nat Rev Neurol.

[62] Kehoe P, Wavrant-De Vrieze F, Crook R, Wu WS, Holmans P, Fenton I, Spurlock G, Norton N, Williams H, Williams N (1999). A full genome scan for late onset Alzheimer’s disease. Hum Mol Genet.

[63] Cezario SM, Calastri MC, Oliveira CI, Carmo TS, Pinhel MA, Godoy MF, Jorge R, Cotrim CC, Souza DR, Siqueira RC (2015). Association of high-density lipoprotein and apolipoprotein E genetic variants with age-related macular degeneration. Arq Bras Oftalmol.

[64] Levy O, Calippe B, Lavalette S, Hu SJ, Raoul W, Dominguez E, Housset M, Paques M, Sahel JA, Bemelmans AP (2015). Apolipoprotein E promotes subretinal mononuclear phagocyte survival and chronic inflammation in age-related macular degeneration. EMBO Mol Med.

[65] Zhang L, Lim SL, Du H, Zhang M, Kozak I, Hannum G, Wang X, Ouyang H, Hughes G, Zhao L (2012). High temperature requirement factor A1 (HTRA1) gene regulates angiogenesis through transforming growth factor-beta family member growth differentiation factor 6. J Biol Chem.

[66] Langton KP, McKie N, Curtis A, Goodship JA, Bond PM, Barker MD, Clarke M (2000). A novel tissue inhibitor of metalloproteinases-3 mutation reveals a common molecular phenotype in Sorsby’s fundus dystrophy. J Biol Chem.

[67] Clarke M, Mitchell KW, Goodship J, McDonnell S, Barker MD, Griffiths ID, McKie N (2001). Clinical features of a novel TIMP-3 mutation causing Sorsby’s fundus dystrophy: implications for disease mechanism. The British journal of ophthalmology.

[68] Fritsche LG, Igl W, Bailey JN, Grassmann F, Sengupta S, Bragg-Gresham JL, Burdon KP, Hebbring SJ, Wen C, Gorski M (2016). A large genome-wide association study of age-related macular degeneration highlights contributions of rare and common variants. Nat Genet.

[69] AREDS (2001). A randomized, placebo-controlled, clinical trial of high-dose supplementation with vitamins C and E, beta carotene, and zinc for age-related macular degeneration and vision loss: AREDS report no. 8. Arch Ophthalmol.

[70] Curcio CA, Johnson M, Huang JD, Rudolf M (2009). Aging, age-related macular degeneration, and the response-to-retention of apolipoprotein B-containing lipoproteins. Progress in retinal and eye research.

[71] Hawkins BS, Bird A, Klein R, West SK (1999). Epidemiology of age-related macular degeneration. Molecular vision.

[72] Khotcharrat R, Patikulsila D, Hanutsaha P, Khiaocham U, Ratanapakorn T, Sutheerawatananonda M, Pannarunothai S (2015). Epidemiology of Age-Related Macular Degeneration among the Elderly Population in Thailand. J Med Assoc Thai.

[73] Kiernan DF, Hariprasad SM, Rusu IM, Mehta SV, Mieler WF, Jager RD (2010). Epidemiology of the association between anticoagulants and intraocular hemorrhage in patients with neovascular age-related macular degeneration. Retina.

[74] Klein R, Peto T, Bird A, Vannewkirk MR (2004). The epidemiology of age-related macular degeneration. American journal of ophthalmology.

[75] Meyers KJ, Liu Z, Millen AE, Iyengar SK, Blodi BA, Johnson E, Snodderly DM, Klein ML, Gehrs KM, Tinker L (2015). Joint Associations of Diet, Lifestyle, and Genes with Age-Related Macular Degeneration. Ophthalmology.

[76] Seddon JM, George S, Rosner B (2006). Cigarette smoking, fish consumption, omega-3 fatty acid intake, and associations with age-related macular degeneration: the US Twin Study of Age-Related Macular Degeneration. Arch Ophthalmol.

[77] Schick T, Ersoy L, Lechanteur YT, Saksens NT, Hoyng CB, den Hollander AI, Kirchhof B, Fauser S (2016). History of Sunlight Exposure Is a Risk Factor for Age-Related Macular Degeneration. Retina.

[78] Millen AE, Meyers KJ, Liu Z, Engelman CD, Wallace RB, LeBlanc ES, Tinker LF, Iyengar SK, Robinson JG, Sarto GE (2015). Association between vitamin D status and age-related macular degeneration by genetic risk. JAMA Ophthalmol.

[79] Binder CJ, Chang MK, Shaw PX, Miller YI, Hartvigsen K, Dewan A, Witztum JL (2002). Innate and acquired immunity in atherogenesis. Nat Med.

[80] Brewer GJ (2007). Iron and copper toxicity in diseases of aging, particularly atherosclerosis and Alzheimer’s disease. Exp Biol Med (Maywood).

[81] Beatty S, Koh H, Phil M, Henson D, Boulton M (2000). The role of oxidative stress in the pathogenesis of age-related macular degeneration. Surv Ophthalmol.

[82] Hollyfield JG, Bonilha VL, Rayborn ME, Yang X, Shadrach KG, Lu L, Ufret RL, Salomon RG, Perez VL (2008). Oxidative damage-induced inflammation initiates age-related macular degeneration. Nat Med.

[83] Kim GH, Kim JE, Rhie SJ, Yoon S (2015). The Role of Oxidative Stress in Neurodegenerative Diseases. Exp Neurobiol.

[84] Smith W, Assink J, Klein R, Mitchell P, Klaver CC, Klein BE, Hofman A, Jensen S, Wang JJ, de Jong PT (2001). Risk factors for age-related macular degeneration: Pooled findings from three continents. Ophthalmology.

[85] Damian J, Pastor R, Armada F, Arias L (2006). [Epidemiology of age-related macular degeneration. Situation in Spain]. Aten Primaria.

[86] Wang YQ, Dong XG (2005). [Progress in the study of epidemiology and etiology of age-related macular degeneration]. Zhonghua Yan Ke Za Zhi.

[87] Bonastre J, Le Pen C, Anderson P, Ganz A, Berto P, Berdeaux G (2002). The epidemiology, economics and quality of life burden of age-related macular degeneration in France, Germany, Italy and the United Kingdom. Eur J Health Econ.

[88] Seddon JM, Chen CA (2004). The epidemiology of age-related macular degeneration. Int Ophthalmol Clin.

[89] Friedman DS, O’Colmain BJ, Munoz B, Tomany SC, McCarty C, de Jong PT, Nemesure B, Mitchell P, Kempen J, and Eye Diseases Prevalence Research, G (2004). Prevalence of age-related macular degeneration in the United States. Arch Ophthalmol.

[90] Solberg Y, Rosner M, Belkin M (1998). The association between cigarette smoking and ocular diseases. Surv Ophthalmol.

[91] Shen JK, Dong A, Hackett SF, Bell WR, Green WR, Campochiaro PA (2007). Oxidative damage in age-related macular degeneration. Histol Histopathol.

[92] Thurman JM, Renner B, Kunchithapautham K, Ferreira VP, Pangburn MK, Ablonczy Z, Tomlinson S, Holers VM, Rohrer B (2009). Oxidative stress renders retinal pigment epithelial cells susceptible to complement-mediated injury. J Biol Chem.

[93] Wu Z, Lauer TW, Sick A, Hackett SF, Campochiaro PA (2007). Oxidative stress modulates complement factor H expression in retinal pigmented epithelial cells by acetylation of FOXO3. J Biol Chem.

[94] Catala A (2011). Lipid peroxidation of membrane phospholipids in the vertebrate retina. Front Biosci (Schol Ed).

[95] Kevany BM, Palczewski K (2010). Phagocytosis of retinal rod and cone photoreceptors. Physiology (Bethesda).

[96] Shaw PX, Horkko S, Chang MK, Curtiss LK, Palinski W, Silverman GJ, Witztum JL (2000). Natural antibodies with the T15 idiotype may act in atherosclerosis, apoptotic clearance, and protective immunity. J Clin Invest.

[97] Jeitner TM, Voloshyna I, Reiss AB (2011). Oxysterol derivatives of cholesterol in neurodegenerative disorders. Curr Med Chem.

[98] Larrayoz IM, Huang JD, Lee JW, Pascual I, Rodriguez IR (2010). 7-ketocholesterol-induced inflammation: involvement of multiple kinase signaling pathways via NFkappaB but independently of reactive oxygen species formation. Investigative ophthalmology & visual science.

[99] Joffre C, Leclere L, Buteau B, Martine L, Cabaret S, Malvitte L, Acar N, Lizard G, Bron A, Creuzot-Garcher C (2007). Oxysterols induced inflammation and oxidation in primary porcine retinal pigment epithelial cells. Current eye research.

[100] Mylonas C, Kouretas D (1999). Lipid peroxidation and tissue damage. In Vivo.

[101] Duryee MJ, Klassen LW, Schaffert CS, Tuma DJ, Hunter CD, Garvin RP, Anderson DR, Thiele GM (2010). Malondialdehyde-acetaldehyde adduct is the dominant epitope after MDA modification of proteins in atherosclerosis. Free radical biology & medicine.

[102] Kaji Y, Usui T, Oshika T, Matsubara M, Yamashita H, Araie M, Murata T, Ishibashi T, Nagai R, Horiuchi S (2000). Advanced glycation end products in diabetic corneas. Invest Ophthalmol Vis Sci.

[103] Handa JT, Verzijl N, Matsunaga H, Aotaki-Keen A, Lutty GA, te Koppele JM, Miyata T, Hjelmeland LM (1999). Increase in the advanced glycation end product pentosidine in Bruch’s membrane with age. Invest Ophthalmol Vis Sci.

[104] Renganathan K, Ebrahem Q, Vasanji A, Gu X, Lu L, Sears J, Salomon RG, Anand-Apte B, Crabb JW (2008). Carboxyethylpyrrole adducts, age-related macular degeneration and neovascularization. Adv Exp Med Biol.

[105] Barja G, Herrero A (2000). Oxidative damage to mitochondrial DNA is inversely related to maximum life span in the heart and brain of mammals. FASEB J.

[106] Dib B, Lin H, Maidana DE, Tian B, Miller JB, Bouzika P, Miller JW, Vavvas DG (2015). Mitochondrial DNA has a pro-inflammatory role in AMD. Biochimica et biophysica acta.

[107] Lin H, Xu H, Liang FQ, Liang H, Gupta P, Havey AN, Boulton ME, Godley BF (2011). Mitochondrial DNA damage and repair in RPE associated with aging and age-related macular degeneration. Invest Ophthalmol Vis Sci.

[108] Wang AL, Lukas TJ, Yuan M, Neufeld AH (2008). Increased mitochondrial DNA damage and down-regulation of DNA repair enzymes in aged rodent retinal pigment epithelium and choroid. Molecular vision.

[109] Terluk MR, Kapphahn RJ, Soukup LM, Gong H, Gallardo C, Montezuma SR, Ferrington DA (2015). Investigating mitochondria as a target for treating age-related macular degeneration. J Neurosci.

[110] Perez VL, Caspi RR (2015). Immune mechanisms in inflammatory and degenerative eye disease. Trends in immunology.

[111] Du H, Sun X, Guma M, Luo J, Ouyang H, Zhang X, Zeng J, Quach J, Nguyen DH, Shaw PX (2013). JNK inhibition reduces apoptosis and neovascularization in a murine model of age-related macular degeneration. Proc Natl Acad Sci U S A.

[112] Horkko S, Binder CJ, Shaw PX, Chang MK, Silverman G, Palinski W, Witztum JL (2000). Immunological responses to oxidized LDL. Free radical biology & medicine.

[113] Berliner JA, Navab M, Fogelman AM, Frank JS, Demer LL, Edwards PA, Watson AD, Lusis AJ (1995). Atherosclerosis: basic mechanisms. Oxidation, inflammation, and genetics. Circulation.

[114] Ebrahimi KB, Handa JT (2011). Lipids, lipoproteins, and age-related macular degeneration. Journal of lipids.

[115] Chou MY, Hartvigsen K, Hansen LF, Fogelstrand L, Shaw PX, Boullier A, Binder CJ, Witztum JL (2008). Oxidation-specific epitopes are important targets of innate immunity. J Intern Med.

[116] Weismann D, Hartvigsen K, Lauer N, Bennett KL, Scholl HP, Charbel Issa P, Cano M, Brandstatter H, Tsimikas S, Skerka C (2011). Complement factor H binds malondialdehyde epitopes and protects from oxidative stress. Nature.

[117] Ambati J, Atkinson JP, Gelfand BD (2013). Immunology of age-related macular degeneration. Nature reviews. Immunology.

[118] de Oliveira Dias JR, Rodrigues EB, Maia M, Magalhaes O, Penha FM, Farah ME (2011). Cytokines in neovascular age-related macular degeneration: fundamentals of targeted combination therapy. The British journal of ophthalmology.

[119] Mo FM, Proia AD, Johnson WH, Cyr D, Lashkari K (2010). Interferon gamma-inducible protein-10 (IP-10) and eotaxin as biomarkers in age-related macular degeneration. Invest Ophthalmol Vis Sci.

[120] Wang JC, Cao S, Wang A, To E, Law G, Gao J, Zhang D, Cui JZ, Matsubara JA (2015). CFH Y402H polymorphism is associated with elevated vitreal GM-CSF and choroidal macrophages in the postmortem human eye. Molecular vision.

[121] Lad EM, Cousins SW, Van Arnam JS, Proia AD (2015). Abundance of infiltrating CD163+ cells in the retina of postmortem eyes with dry and neovascular age-related macular degeneration. Graefes Arch Clin Exp Ophthalmol.

[122] Doyle SL, Campbell M, Ozaki E, Salomon RG, Mori A, Kenna PF, Farrar GJ, Kiang AS, Humphries MM, Lavelle EC (2012). NLRP3 has a protective role in age-related macular degeneration through the induction of IL-18 by drusen components. Nat Med.

[123] Doyle SL, Lopez FJ, Celkova L, Brennan K, Mulfaul K, Ozaki E, Kenna PF, Kurali E, Hudson N, Doggett T (2015). IL-18 Immunotherapy for Neovascular AMD: Tolerability and Efficacy in Nonhuman Primates. Invest Ophthalmol Vis Sci.

[124] Doyle SL, Ozaki E, Brennan K, Humphries MM, Mulfaul K, Keaney J, Kenna PF, Maminishkis A, Kiang AS, Saunders SP (2014). IL-18 attenuates experimental choroidal neovascularization as a potential therapy for wet age-related macular degeneration. Sci Transl Med.

[125] Skerka C, Zipfel PF (2008). Complement factor H related proteins in immune diseases. Vaccine.

[126] Kelly J, Ali Khan A, Yin J, Ferguson TA, Apte RS (2007). Senescence regulates macrophage activation and angiogenic fate at sites of tissue injury in mice. J Clin Invest.

[127] Xu H, Chen M, Forrester JV (2009). Para-inflammation in the aging retina. Prog Retin Eye Res.

[128] Anderson DH, Radeke MJ, Gallo NB, Chapin EA, Johnson PT, Curletti CR, Hancox LS, Hu J, Ebright JN, Malek G (2010). The pivotal role of the complement system in aging and age-related macular degeneration: hypothesis re-visited. Prog Retin Eye Res.

[129] Crabb JW, Miyagi M, Gu X, Shadrach K, West KA, Sakaguchi H, Kamei M, Hasan A, Yan L, Rayborn ME (2002). Drusen proteome analysis: an approach to the etiology of age-related macular degeneration. Proc Natl Acad Sci U S A.

[130] Yuan X, Gu X, Crabb JS, Yue X, Shadrach K, Hollyfield JG, Crabb JW (2010). Quantitative proteomics: comparison of the macular Bruch membrane/choroid complex from age-related macular degeneration and normal eyes. Mol Cell Proteomics.

[131] Johnson LV, Ozaki S, Staples MK, Erickson PA, Anderson DH (2000). A potential role for immune complex pathogenesis in drusen formation. Exp Eye Res.

[132] Wang L, Clark ME, Crossman DK, Kojima K, Messinger JD, Mobley JA, Curcio CA (2010). Abundant lipid and protein components of drusen. PLoS One.

[133] Vierkotten S, Muether PS, Fauser S (2011). Overexpression of HTRA1 leads to ultrastructural changes in the elastic layer of Bruch’s membrane via cleavage of extracellular matrix components. PLoS One.

[134] Skeie J, Mullins R (2009). Macrophages in neovascular age-related macular degeneration: friends or foes?. Eye.

[135] Killingsworth M, Sarks J, Sarks S (1990). Macrophages related to Bruch’s membrane in age-related macular degeneration. Eye.

[136] Combadiere C, Feumi C, Raoul W, Keller N, Rodéro M, Pézard A, Lavalette S, Houssier M, Jonet L, Picard E (2007). CX3CR1-dependent subretinal microglia cell accumulation is associated with cardinal features of age-related macular degeneration. The Journal of Clinical Investigation.

[137] Liang KJ, Lee JE, Wang YD, Ma W, Fontainhas AM, Fariss RN, Wong WT (2009). Regulation of dynamic behavior of retinal microglia by CX3CR1 signaling. Investigative ophthalmology & visual science.

[138] Grossniklaus H, Ling J, Wallace T, Dithmar S, Lawson D, Cohen C, Elner V, Elner S, Sternberg P (2002). Macrophage and retinal pigment epithelium expression of angiogenic cytokines in choroidal neovascularization. Molecular vision.

[139] Yamada K, Sakurai E, Itaya M, Yamasaki S, Ogura Y (2007). Inhibition of Laser-Induced Choroidal Neovascularization by Atorvastatin by Downregulation of Monocyte Chemotactic Protein-1 Synthesis in Mice. Investigative ophthalmology & visual science.

[140] Mettu PS, Wielgus AR, Ong SS, Cousins SW (2012). Retinal pigment epithelium response to oxidant injury in the pathogenesis of early age-related macular degeneration. Molecular aspects of medicine.

[141] Sica A, Mantovani A (2012). Macrophage plasticity and polarization: in vivo veritas. The Journal of Clinical Investigation.

[142] Cherepanoff S, McMenamin P, Gillies MC, Kettle E, Sarks SH (2010). Bruch’s membrane and choroidal macrophages in early and advanced age-related macular degeneration. British Journal of Ophthalmology.

[143] Sindrilaru A, Peters T, Wieschalka S, Baican A, Peter H, Hainzl A, Schatz S, Schlecht S, Qi Y, Schlecht A An unrestrained proinflammatory M1 macrophage population induced by iron impairs wound healing in humans and mice. The Journal of Clinical Investigation.

